# Determinants of disagreement with female genital mutilation/cutting of future daughters and awareness of the ban among Egyptian university students

**DOI:** 10.1186/s12978-020-00941-8

**Published:** 2020-06-10

**Authors:** Aya Mostafa, Shaimaa Ashmawy Gaballah, Ghada Essamaldin Amin

**Affiliations:** 1 Department of Community, Environmental and Occupational Medicine, Faculty of Medicine, Ain Shams University, Abbasia Square, PO-box 11566, Cairo, Egypt; 2 Family Medicine Unit, Menoufia, Egypt

**Keywords:** Female genital mutilation/cutting, University students, Gender, Knowledge, Perception, Policy, Ban, Egypt

## Abstract

**Background:**

Egypt is one of three countries where half of female genital mutilation/cutting (FGM/C) victims live, despite its ban. To inform policy on the awareness of this ban and the impact of other interventions, this study sought to assess FGM/C-related knowledge, perceptions, and determinants of disagreement with FGM/C and circumcision of future daughters among university students.

**Methods:**

A cross-sectional study was conducted using a self-administered questionnaire in a random sample of 502 male and female students in Menoufia University between September and December 2017. Bivariate and multivariable logistic regression analyses were performed.

**Results:**

Students were 21.0 ± 1.6 years old; 270 (54.0%) were males, 291 (58.0%) were non-medical students, and 292 (58.2%) were rural residents. 204 (46.7%) students were not aware of the ban and their main source of information about FGM/C was educational curricula or health education sessions (162, 37.0%). Only 95 (19.0%) students had good knowledge about FGM/C. 217 (43.3%) students were neutral towards discontinuing FGM/C. 280 (56.2%) students disagreed with FGM/C. 296 (59.3%) students disagreed with circumcision of their future daughters; independent determinants of this outcome were awareness of the ban (ORa = 1.9) and disagreement with: FGM/C preserves females’ virginity (ORa = 5.0), has religious basis (ORa = 3.8), makes females happier in marriage (ORa = 3.5), enhances females’ hygiene (ORa = 2.1).

**Conclusions:**

Knowledge about FGM/C and its ban is low, even in this educated population. FGM/C is still misperceived as a religious percept. Maximizing the utilization of health education and curricula might help increase anti-FGM/C attitudes among university students with neutral perceptions and initiate the much-needed momentum for elimination.

## Plain English summary

The United Nations has set a target to eliminate FGM/C by 2030. FGM/C affects at least 200 million girls and women worldwide. Some studies reported that men also suffer from its consequences. Egypt is one of three countries where half of FGM/C victims live. Despite its complete ban in Egypt since 2008, FGM/C is still practiced. To embark on ending FGM/C in one decade, understanding both male and female youth’s perspectives becomes vital in creating a rapid and sustained momentum for elimination.

This study is the first to inform policy on the awareness of this ban and the impact of other interventions among university students and assess FGM/C-related knowledge, perceptions, and determinants of disagreement with FGM/C and circumcision of future daughters. Alarmingly, almost half of the students were not aware of the FGM/C ban. There was a universal lack of good knowledge about FGM/C not only in non-medical, but even in medical students. FGM/C was still misperceived as a religious percept. There was a wide base (43.3%) of neutral perceptions about FGM/C, which might hinder the progress towards elimination. However, this finding may present itself as an opportunity to intensify targeted engagement and education efforts promoting the abandonment of FGM/C among students with neutral perceptions. Also, this study highlighted opportunities for potential improvement: the main source of students’ knowledge about FGM/C was university curricula and health education sessions, therefore, utilizing these potential gateways is crucial to initiate the much-needed momentum for elimination.

## Background

Female genital mutilation/cutting (FGM/C) is a worldwide public health and human rights issue. As an international response, the fifth United Nations’ Sustainable Development Goal included a target to eliminate the practice by 2030 [1]. FGM/C refers to traditional practices involving “partial or total excision of the female external genitalia for non-medical purposes” [2]. FGM/C not only results in short- and long-term physical and psychological complications, but leads to death in some girls [2]. Families have been increasingly seeking medical instead of traditional practitioners for their daughters’ circumcision to minimize pain and complications, while meeting the cultural demand [3]. The World Health Organization has condemned this ‘medicalization’ of the practice because it perpetuates FGM/C rather than abolishes it [4].

FGM/C victims live mainly in Africa and Asia [5], and due to immigration, some live in Europe, Australia, the UK and the USA, where FGM/C and its complications have become an issue of growing concern [6, 7]. At least half of the globally estimated 200 million girls and women who have undergone FGM/C live in only three countries: Indonesia, Ethiopia, and Egypt [5]. In Egypt, 92.3% of ever married women (15–49 years old) and 21.3% of their daughters (0–19 years old) have been subjected to FGM/C, according to the latest Demographic Health Survey in 2015 [8]. Moreover, 58% of Egyptian women believed the practice should continue, although this support has declined by 24% over the last two decades [8]. This is despite Egypt has banned FGM/C in 2008 [9]. There are various reasons for performing FGM/C. In 2008, circumcised Egyptian schoolgirls have reported cleanliness, culture, chastity, and most commonly religious precepts, as reasons that support the continuation of the practice [10]. In 2015, more than half of Egyptian women have similarly reported it on religious basis [8], although several fatwas from religious leaders have been issued to counter these misconceptions in Egypt and in other countries [11].

However, a significant change is unlikely if the ban is implemented in isolation of other interventions (such as health education), considering the deeply rooted public misbeliefs [12, 13]. Also, the extent of awareness of the ban and the impact of other interventions are unknown. Younger generations with higher education would be presumably less supportive of FGM/C, more likely to have been exposed to such interventions, and would likely lead future change and advocate to break this community norm, but have been scarcely studied in Egypt [14, 15] and elsewhere [16–18].

To inform FGM/C prevention policy on the awareness of the FGM/C ban and the impact of other interventions, this study sought to assess and compare medical and non-medical university students’ FGM/C-related knowledge, perceptions, and determinants of disagreement with FGM/C in general and circumcision of their future daughters in particular.

## Methods

### Study design and setting

A cross-sectional study was conducted between September and December 2017 in a public university in Menoufia governorate in the Nile Delta of Egypt. Menoufia has urban and rural localities and has the second highest prevalence of FGM/C in daughters 0–19 years old among the Nile Delta governorates [8].

### Study sample and sampling procedure

The target population included male and female university students (approximately 80,000) attending different faculties of Menoufia University. There are 17 faculties in Menoufia University, from which we randomly selected 7 faculties representing medical and non-medical specialties. The selected faculties representing medical specialties included: Medicine, Pharmacy and Nursing, while those representing non-medical specialties included: Law, Commerce, Engineering and Science. University students who were in their fourth year of studies (approximately 9700 students) were targeted being the final year of studying for non-medical specialties. A sample size of 493 university students with a sample proportion 1:1 from medical and non-medical students was calculated at a 95% level of confidence, a study power of 80%, and an alpha error of 5%, under the hypothesis that 28% of medical students would object to FGM/C abolishment [14] and that proportion would be 10% higher among non-medical students. Another approximately 2% of this required sample size (n = 9) was added to account for possible non-response and missing data; the target sample was 502 university students.

One trained field interviewer visited each faculty on two alternating days per week for data collection. On the day of data collection, the list of practical classes of fourth year students on that day was obtained, from which one class was randomly selected. Approximately 40 students attended the class in each of the selected faculties. After the class, the field interviewer approached the attending students and asked whether they were interested to participate in the study. Data collection was conducted until the target sample size was achieved.

### Study tool and data collection

After obtaining verbal consent, participants filled an anonymous self-administrated structured questionnaire in Arabic language that took approximately 15 min to complete. The questionnaire items were adapted from previous literature [8, 14] and were pretested on 40 students from Faculties of Medicine and Science for clarity of the questions and the answer categories; pretest data are not included in this analysis. The questionnaire consisted of three sections:

1. Socio-demographic characteristics: age, gender, rural/urban residence, parents’ educational attainment, and faculty.
2. Students’ knowledge about FGM/C (4 questions): whether they have any information about FGM/C (yes/no); the main source of their information about FGM/C (family/mass media/educational curricula studied in the university/ health education seminars/other); the purpose of practicing FGM/C (more than one answer option was allowed: traditions/religious/medical/cosmetic/hygienic/facilitation of easy delivery in the future/male sexual satisfaction/assurance of virginity); and whether they were aware of any legislation that bans FGM/C in Egypt (yes/no).
3. Students’ perception regarding FGM/C (12 items): students answered a 5-likert scale for each item whether they: “strongly agree” (1), “agree” (2), “neutral” (3), “disagree” (4), “strongly disagree” (5) with: acceptance of the FGM/C practice; females should perform FGM/C before marriage; FGM/C should be banned from media discussions; the television is unimportant in preventing FGM/C; FGM/C has religious basis; females are indecent until they are circumcised; circumcised females are happier in their marital lives; females cannot please their husbands if uncircumcised; FGM/C enhances females’ personal hygiene; FGM/C preserves females’ virginity; circumcised females are more respected by the community; and my future daughters should be circumcised.

### Statistical analysis

Serial identification numbers were assigned to each anonymously filled questionnaire. Data were analysed using SPSS (Statistical Package for the Social Sciences, version 25, SSPS Inc., Chicago, IL, USA). Correct knowledge was considered if the student answered “yes” to the (yes/no) knowledge questions, answered “educational curricula” or “health education” or “mass media” to the source of knowledge question, and “traditions” or “medical” to the purpose of FGM/C question. For each correct answer, the participant was assigned a score of 1 and otherwise 0. Positive perception (favourable response towards discontinuation of FGM/C) was considered if the participant responded “disagree” or “strongly disagree” to the 12 perception items. For each participant, a total knowledge and perception percentage score was calculated. Then, mean percentage scores were calculated and categorized into < 50%, 50–75, and > 75%, representing “poor”, “average”, and “good” knowledge, or “negative”, “neutral”, and “positive” perception. Descriptive statistics were performed and presented as frequency and percentages for qualitative variables or mean and standard deviation for quantitative variables. Bivariate analyses were performed using the Chi-squared test or the Independent Samples T-test. Multivariable logistic regression analyses were used to identify the factors associated with disagreement with the practice of FGM/C and circumcision of future daughters among university students, testing the following variables as independent determinants: female gender, urban residence, higher parental education, students of medical faculties, educational curricula or health education as the main source of knowledge about FGM/C, consideration of FGM/C only for a medical purpose, awareness of a legislation that bans FGM/C, and disagreement with the perception items. Adjusted odds ratios (ORa) and 95% confidence intervals (CI) are reported. A p-value ≤0.05 was considered statistically significant.

### Ethical considerations

The Research Ethics Committee, Faculty of Medicine, Ain Shams University, Cairo, Egypt approved this study. Menoufia University provided permission for conduction of the study in its premises. Potential participants were informed about the study objectives and were assured about confidentiality and anonymity of their responses, that their participation was voluntary, and their freedom to withdraw from the study at any time. Students provided consent prior to questionnaire completion.

## Results

### Sample characteristics

A total of 502 university students attending Menoufia University participated in the present study: 211 (42.0%) from faculties representing medical specialties (Medicine *n* = 49, Pharmacy *n* = 78, and Nursing *n* = 84), and 291 (58.0%) from faculties representing non-medical specialties (Law *n* = 51, Commerce *n* = 117, Engineering *n* = 59, and Science *n* = 63). The students’ mean age was 21.0 ± 1.6 years. More than half of the students were males (270, 54.0%), and lived in rural areas (292, 58.2%). Approximately half of the students` mothers (236, 47.1%) and fathers (265, 53.2%) had completed secondary or a higher education (Table 1). Sample characteristics of students in the three faculties representing medical specialities as well as the four faculties representing non-medical specialities are described in Supplementary Table 1.

**Table 1:** Sample characteristics, knowledge and perceptions about FGM/C among medical and non-medical university students (*n* = 502)

	Total^**a**^		Non-medical		Medical		χ^**2**^	***p***-value^**c**^
	*N* = 502	%	*N* = 291	%	*N* = 211	%		
**Gender**, male	270	54.0	164	56.6	106	50.5	1.810	0.203
**Residence**, rural	292	58.2	167	57.4	125	59.2	0.173	0.714
**Mother’s education**								
Secondary complete/higher	236	47.1	126	43.4	110	52.1	5.327	0.070
Primary complete/some secondary	201	40.1	120	41.4	81	38.4		
No education/some primary	64	12.8	44	15.2	20	9.5		
**Father’s education**								
Secondary complete/higher	265	53.2	146	50.5	119	56.9	5.485	0.064
Primary complete/some secondary	209	42.0	124	42.9	85	40.7		
No education/some primary	24	4.8	19	6.6	5	2.4		
**Knows about FGM/C**, yes	440	88.7	247	86.7	193	91.5	2.792	0.114
**Aware of any legislation that bans FGM/C**, yes	204	46.7	111	45.1	93	48.7	0.55	0.499
**Source of knowledge about FGM/C**								
Family	140	32.0	91	37.0	49	25.5	8.714	0.033
Educational curricula/health education	162	37.0	89	36.2	73	38.0		
Mass media	90	20.5	41	16.7	49	25.5		
Other	46	10.5	25	10.2	21	10.9		
**Purpose of conducting FGM/C**^b^								
Traditions	372	67.9	200	64.5	172	72.3	7.468	0.058
Religious	95	17.3	52	16.8	43	18.1		
Medical	22	4.0	15	4.8	7	2.9		
Other	59	10.8	43	13.9	16	6.7		
**Disagree with perception items:**								
Acceptance of the FGM/C practice	280	56.2	151	52.6	129	61.1	3.590	0.068
Females should be circumcised before marriage	296	59.3	158	54.7	138	65.7	6.146	0.016
FGM/C should be banned from media discussions	337	67.8	188	65.5	149	71.0	1.648	0.208
The television is unimportant in preventing FGM/C	324	65.1	184	63.9	140	66.7	0.412	0.568
FGM/C has religious basis	198	39.7	106	36.6	92	44.0	2.830	0.096
Females are indecent until they are circumcised	361	72.3	202	69.7	159	76.1	2.503	0.128
Circumcised females are happier in their marital lives	280	56.2	148	51.0	132	63.5	7.600	0.006
Females cannot please their husbands if uncircumcised	315	63.3	171	59.0	144	69.2	5.491	0.024
FMG/C enhances females’ personal hygiene	276	55.4	157	54.1	119	57.2	0.463	0.523
FMG/C preserves females’ virginity	283	56.8	157	54.1	126	60.6	2.047	0.169
Circumcised females are more respected by community	338	67.7	192	66.2	146	69.9	0.740	0.438
My future daughters should be circumcised	296	59.3	163	56.2	133	63.3	2.778	0.098

*FGM/C* Female genital mutilation/cutting

^a^ Some variables had missing values

^b^ More than one answer option was allowed

^c^ Chi-squared test

### Knowledge of university students about FGM/C

Most of the students knew about FGM/C (440, 88.7%); among whom almost a half (204, 46.7%) were not aware of legislations that ban FGM/C. The main source of these students’ information about FGM/C was the educational curricula they studied at university or health education sessions (162, 37.0%), followed by the family (140, 32.0%), and mass media (90, 20.5%). A significantly larger proportion of non-medical than medical students and females than males relied mainly on their family as the main source of information about FGM/C. More than two-thirds of the students (372, 67.9%) reported that the purpose of conducting FGM/C was traditions, 95 (17.3%) reported it has religious purposes, and 22 (4.0%) reported it has medical purposes (Tables 1 and 2).

**Table 2:** Sample characteristics, knowledge and perceptions about FGM/C among male and female university students (*n* = 502)

	Total^**a**^		Males		Females		χ^**2**^	***p***-value^**c**^
	*N* = 500	%	*N* = 270	%	*N* = 230	%		
**Faculty**, non-medical	290	58.0	164	60.7	126	54.8	1.810	0.203
**Residence**, rural	290	58.0	156	57.8	134	58.3	0.012	0.928
**Mother’s education**								
Secondary complete/higher	236	47.3	131	48.7	105	45.7	1.768	0.413
Primary complete/some secondary	200	40.1	101	37.5	99	43.0		
No education/some primary	63	12.6	37	13.8	26	11.3		
**Father’s education**								
Secondary complete/higher	264	53.2	135	50.4	129	56.6	2.484	0.289
Primary complete/some secondary	208	41.9	121	45.1	87	38.2		
No education/some primary	24	4.8	12	4.5	12	5.3		
**Knows about FGM/C**, yes	438	88.7	237	88.8	201	88.5	0.006	1.000
**Aware of any legislation that bans FGM/C**, yes	202	46.4	105	44.5	97	48.7	0.785	0.387
**Source of knowledge about FGM/C**								
Family	139	31.9	61	25.8	78	39.0	9.779	0.021
Educational curricula/health education	161	36.9	64	32.0	97	41.1		
Mass media	90	20.6	49	20.8	41	20.5		
Other	46	10.6	29	12.3	17	8.5		
**Purpose of conducting FGM/C**^b^								
Traditions	353	64.8	191	65.0	180	71.7	3.247	0.355
Religious	94	17.2	53	18.0	41	16.3		
Medical	21	3.9	14	4.8	7	2.8		
Other	59	10.8	36	12.2	23	9.2		
**Disagree with perception items:**								
Acceptance of the FGM/C practice	279	56.3	141	52.4	138	60.8	3.510	0.069
Females should be circumcised before marriage	295	59.4	153	56.9	142	62.3	1.493	0.234
FGM/C should be banned from media discussions	336	67.9	180	67.4	156	68.4	0.057	0.847
The television is unimportant in preventing FGM/C	323	65.1	168	62.7	155	68.0	1.521	0.221
FGM/C has religious basis	197	39.6	102	44.7	95	35.3	4.578	0.035
Females are indecent until they are circumcised	359	72.2	179	78.5	180	66.9	8.271	0.005
Circumcised females are happier in their marital lives	278	56.0	140	52.0	138	60.8	3.825	0.057
Females cannot please their husbands if uncircumcised	313	63.1	154	67.8	159	59.1	4.033	0.050
FMG/C enhances females’ personal hygiene	274	55.2	132	49.1	142	62.6	9.054	0.003
FMG/C preserves females’ virginity	282	56.9	132	49.1	150	66.1	14.519	< 0.001
Circumcised females are more respected by community	336	67.6	172	63.9	164	71.9	3.597	0.068
My future daughters should be circumcised	295	59.4	145	53.9	150	65.8	7.227	0.008

*FGM/C* Female genital mutilation/cutting

^a^ Some variables had missing values

^b^ More than one answer option was allowed

^c^ Chi-squared test

### Perception of university students regarding FGM/C

**Fig. 1:**
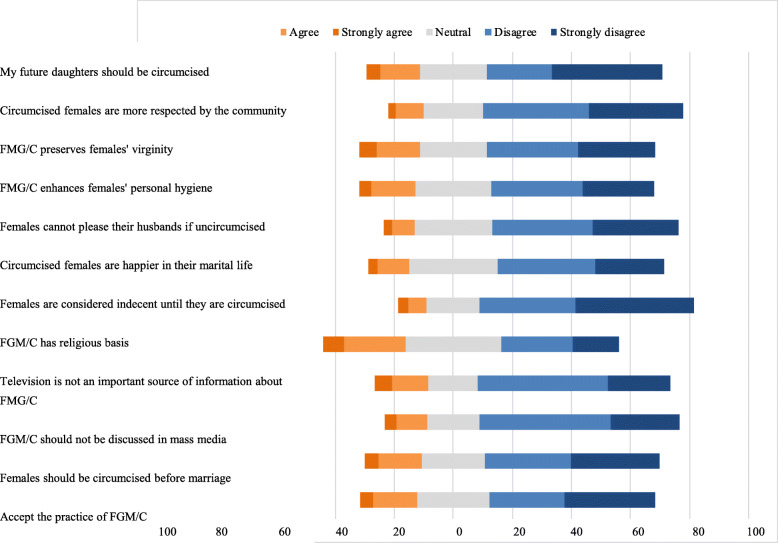
Percentage of university students who ___________ with the following perception items

Overall responses to perception items are presented in Fig. 1. In general, more than a half of the students did not accept the practice of FGM/C (280, 56.2%) and disagreed with their future daughters being circumcised (296, 59.3%). More than a half to approximately two-thirds of the students disagreed with all the perception items. Approximately two-fifths (198, 39.7%) disagreed with the item FGM/C has religious basis. Significantly more medical than non-medical students disagreed with the following perception items: females should perform FGM/C before marriage (65.7% versus 54.7%); circumcised females are happier in their marital lives (63.5% versus 51.0%); and females cannot please their husbands if uncircumcised (69.2% versus 59.0%). Significantly more males than females disagreed with the items: FGM/C has religious basis (44.7% versus 35.3%) and females are indecent until they are circumcised (78.5% versus 66.9%). Significantly more females than males disagreed with the following perception items: FMG/C enhances females’ personal hygiene (62.6% versus 49.1%); FMG/C preserves females’ virginity (66.1% versus 49.1%); and my future daughters should be circumcised (65.8% versus 53.9%) (Tables 1 and 2).

### Mean total percentage scores and levels of knowledge and perception about FGM/C

Students’ mean total percentage scores were below 75. Approximately one-fifth (95, 19.0%) of the students had a good level of knowledge. Approximately half of the students (238, 47.5%) reported positive perception and 217 (43.3%) reported neutral perception towards discontinuing the practice of FGM/C. Differences between medical/non-medical students, males/females, urban/rural residents, and by level of parental education are presented in Table 3.

**Table 3:** Mean total percentage scores and levels of knowledge and perception about FGM/C among university students (*n* = 502)

		Mean total percentage score of knowledge	Mean total percentage score of perception	Level of knowledge n (%)			Level of perception n (%)		
Characteristic	N^**a**^	mean (SD)	mean (SD)	Poor	Average	Good	Negative	Neutral	Positive
**Total**	501	66.3 (23.9)	72.9 (16.2)	68 (13.6)	338 (67.5)	95 (19.0)	46 (9.2)	217 (43.3)	238 (47.5)
**Faculty**									
Non-medical	290	63.2 (23.9)	71.6 (15.3)	48 (16.6)	201 (69.3)	41 (14.1)	25 (8.6)	142 (49.0)	123 (42.4)
Medical	211	70.5 (23.1)	74.7 (17.3)	20 (9.5)	137 (64.5)	54 (25.6)	21 (10.0)	75 (35.5)	115 (54.5)
Statistic^b^		2.109	3.041	13.300			9.072		
*p*-value		0.001	0.032	0.001			0.011		
**Gender**									
Males	270	65.9 (24.9)	70.8 (15.8)	43 (15.9)	171 (63.3)	56 (20.7)	26 (9.6)	136 (50.4)	108 (40.0)
Females	229	66.5 (22.5)	75.4 (16.4)	25 (10.9)	166 (72.5)	38 (16.6)	20 (8.7)	80 (34.9)	129 (56.3)
Statistic^b^		4.617	0.207	4.950			13.887		
*p*-value		0.795	0.002	0.084			0.001		
**Residence**									
Rural	292	65.3 (23.7)	70.7 (16.9)	40 (13.7)	202 (69.2)	50 (17.1)	37 (12.7)	131 (44.9)	124 (42.5)
Urban	209	67.9 (24.1)	75.9 (14.7)	28 (13.4)	136 (65.1)	45 (21.5)	9 (4.3)	86 (41.1)	114 (54.5)
Statistic^b^		0.008	6.906	1.561			13.413		
*p*-value		0.297	< 0.001	0.458			0.001		
**Mother’s education**									
Completed secondary or higher	235	69.7 (23.5)	74.9 (16.4)	24 (10.2)	155 (66.0)	56 (23.8)	19 (8.1)	89 (37.9)	127 (54.0)
Other	265	63.5 (23.5)	71.2 (15.9)	43 (16.2)	183 (69.1)	39 (14.7)	27 (10.2)	127 (47.9)	111 (41.9)
Statistic^b^		1.226	0.268	8.982			7.379		
*p*-value		0.003	0.010	0.011			0.025		
**Father’s education**									
Completed secondary or higher	265	68.2 (24.3)	74.4 (16.9)	31 (11.7)	173 (65.3)	61 (23.0)	21 (7.9)	106 (40.0)	138 (52.1)
Other	232	63.7 (23.2)	71.3 (15.4)	37 (15.9)	163 (70.3)	32 (13.8)	25 (10.8)	109 (47.0)	98 (42.2)
Statistic^b^		0.017	3.778	7.713			5.000		
*p*-value		0.035	0.032	0.021			0.082		

*FGM/C* Female genital mutilation/cutting

^a^ Some variables had missing values

^b^ F statistic for Independent Samples t-test and χ^2^ statistic for Chi-squared test

### Determinants of disagreement with the practice of FGM/C and circumcision of future daughters

Of the variables tested in the multivariable logistic regression model, the following were independent determinants of disagreement with the practice of FGM/C: urban residence (ORa = 3.6, 95%CI:1.9–6.7), and disagreement with the following perception items: ‘FGM/C has religious basis’ (ORa = 6.3, 95%CI:3.3–12.0), ‘FMG/C preserves females’ virginity’ (ORa = 3.8, 95%CI:1.9–7.7), ‘circumcised females are happier in their marital lives’ (ORa = 3.7, 95%CI:1.9–7.2), ‘females are indecent until they are circumcised’ (ORa = 2.5, 95%CI:1.2–5.4), and ‘the television is unimportant in preventing FGM/C’ (ORa = 2.1, 95%CI:1.1–3.8). The following variables were independent determinants of disagreement with circumcision of future daughters: awareness of a legislation that bans FGM/C (ORa = 1.9, 95%CI:1.1–3.4), and disagreement with the following perception items: ‘FMG/C preserves females’ virginity’ (ORa = 5.0, 95%CI:2.5–9.9), ‘FGM/C has religious basis’ (ORa = 3.8, 95%CI:1.9–7.6), ‘circumcised females are happier in their marital lives’ (ORa = 3.5, 95%CI:1.8–6.9), ‘FMG/C enhances females’ personal hygiene’ (ORa = 2.1, 95%CI:1.1–4.4), and ‘the television is unimportant in preventing FGM/C’ (ORa = 2.1, 95%CI:1.2–3.9) (Table 4). The perception item ‘females should be circumcised before marriage’ was not included in the multivariable model because it showed high correlation with the dependent variables.

**Table 4:** Factors associated with disagreement with the practice of FGM/C and circumcision of future daughters among university students (*n* = 502)

Characteristic	Disagree with the practice of FGM/C				Disagree with circumcision of future daughters			
	Unadjusted OR^**a**^ (95% CI)	***p***-value	Adjusted OR^**b**^ (95% CI)	***p***-value	Unadjusted OR^**a**^ (95% CI)	***p***-value	Adjusted OR^**b**^ (95% CI)	***p***-value
**Gender** (female vs male)	1.4 (0.9–2.0)	0.069	0.9 (0.5–1.6)	0.788	1.7 (1.1–2.4)	0.008	1.1 (0.6–1.9)	0.737
**Residence** (urban vs rural)	2.1 (1.5–3.1)	< 0.001	3.6 (1.9–6.7)	< 0.001	1.5 (1.1–2.2)	0.027	1.8 (0.9–3.3)	0.051
**Mother education** (completed secondary or higher vs other)	1.4 (0.9–1.9)	0.086	1.2 (0.6–2.3)	0.581	1.9 (1.3–2.7)	0.001	1.8 (0.9–3.4)	0.126
**Father education** (completed secondary or higher vs other)	1.2 (0.9–1.7)	0.317	0.7 (0.4–1.4)	0.328	1.5 (1.1–2.2)	0.022	1.1 (0.6–2.3)	0.707
**Faculty** (medical vs non-medical)	1.4 (0.9–2.0)	0.068	1.2 (0.7–2.0)	0.575	1.4 (0.9–1.9)	0.098	1.1 (0.6–1.9)	0.765
**Main source of knowledge about FGM/C** (educational curricula/health education vs other)	1.3 (0.9–1.9)	0.206	1.4 (0.8–2.5)	0.257	1.0 (0.7–1.5)	1.000	0.8 (0.4–1.4)	0.358
**Main purpose of FGM/C** (only medical purpose vs other)	0.9 (0.4–2.2)	1.000	1.6 (0.5–5.3)	0.461	0.5 (0.2–1.1)	0.079	0.4 (0.1–1.5)	0.184
**Aware of a legislation that bans FGM/C** (yes vs no)	1.5 (1.1–2.2)	0.023	0.8 (0.5–1.5)	0.550	2.4 (1.6–3.5)	< 0.001	1.9 (1.1–3.4)	0.029
**Disagrees with the following perception items** (vs agrees):								
FGM/C should be banned from media discussions	2.3 (1.6–3.3)	< 0.001	1.0 (0.5–1.9)	0.963	2.2 (1.5–3.3)	< 0.001	0.8 (0.4–1.5)	0.498
The television is unimportant in preventing FGM/C	4.4 (2.9–6.6)	< 0.001	2.1 (1.1–3.8)	0.017	4.8 (3.3–7.2)	< 0.001	2.1 (1.2–3.9)	0.014
FGM/C has religious basis	9.3 (5.9–14.6)	< 0.001	6.3 (3.3–12.0)	< 0.001	7.5 (4.8–11.8)	< 0.001	3.8 (1.9–7.6)	< 0.001
Females are indecent until they are circumcised	6.2 (3.9–9.7)	< 0.001	2.5 (1.2–5.4)	0.019	6.3 (4.0–9.7)	< 0.001	1.8 (0.9–3.7)	0.115
Circumcised females are happier in their marital lives	10.9 (7.2–16.6)	< 0.001	3.7 (1.9–7.2)	< 0.001	10.8 (7.1–16.5)	< 0.001	3.5 (1.8–6.9)	< 0.001
Females cannot please their husbands if uncircumcised	4.3 (2.9–6.4)	< 0.001	0.7 (0.3–1.3)	0.243	3.9 (2.6–5.7)	< 0.001	0.6 (0.3–1.2)	0.146
FMG/C enhances females’ personal hygiene	9.5 (6.3–14.4)	< 0.001	1.7 (0.9–3.3)	0.099	11.5 (7.5–17.6)	< 0.001	2.1 (1.1–4.1)	0.025
FMG/C preserves females’ virginity	14.1 (9.4–21.9)	< 0.001	3.8 (1.9–7.7)	< 0.001	18.0 (11.5–28.4)	< 0.001	5.0 (2.5–9.9)	< 0.001
Circumcised females are more respected by community	5.1 (3.4–7.6)	< 0.001	0.7 (0.4–1.5)	0.388	6.2 (4.1–9.4)	< 0.001	0.9 (0.5–1.9)	0.914

*FGM/C* Female genital mutilation/cutting, *OR* Odds ratio, *CI* confidence interval

^a^ Chi-squared test

^b^ Multivariable logistic regression

## Discussion

Approximately half (46.7%) of the university students in this study were not aware of the ban on FGM/C in Egypt. This alarming finding reflects the failure of awareness raising campaigns in communicating a fundamental milestone in combating the practice. We could not find any published results of studies that have directly investigated the awareness of the ban, yet this is an integral message that should be incorporated in future efforts to support the abolishment of FGM/C. Only a fifth (19.0%) of the students had good knowledge about FGM/C. This proportion was significantly higher among medical (25.6%) than non-medical students (14.6%), but the difference was small. This finding suggests that lack of knowledge is general. However, students’ main source of information about FGM/C was educational curricula or health education sessions (37.0%). Investing in research that informs on the best approaches for maximizing the utilization of university educational curricula and health education sessions as potential gateways to universal exposure to knowledge about FGM/C is crucial to initiate the much-needed momentum for elimination of FGM/C.

While more than half of the students disagreed with FGM/C (56.2%) and with their future daughters being circumcised (59.3%), a considerable proportion (43.3%) of students were neutral about discontinuing FGM/C. This is another interesting finding; neutral perceptions about FGM/C do not only form a challenge that may weaken and delay the overall progress towards elimination, they are unacceptable from human rights and public health perspectives. Formulating messages that convey the worldwide documented complications of the procedure and resultant mortalities among girls and women [2] can significantly affect this wide base of neutral perceptions. This finding may present itself as a golden opportunity to intensify targeted engagement and education efforts promoting the abandonment of FGM/C. The proportion agreeing with the practice has decreased by only 10% from a similar local study conducted two decades earlier [14]. Also, there has been only a modest observed reduction in prevalence of and attitudes about FGM/C comparing the situation before and after the ban [9, 19]. Interventions regarding FGM/C have been sporadic; the evaluation of their impact has not been systematically documented [12, 20]. Few local studies in Upper Egypt have indirectly investigated the impact of the FGM/C ban. Rasheed et al. reported that the annual incidence of FGM/C in the 2 years post- implementation of the ban was still ‘very high’ (7%) among girls and young women [21]. The authors also report that 34.4% of young physicians approved the practice; a proportion similar to that found among medical students in the present study (38.4%). Hassanin et al. examined the prevalence of FGM/C among girls after 6 years of implementation of the ban and found that the practice is high and the authors called for revising public awareness and changing attitudes [22]. This modest change in prevalence despite the implementation may be explained by our finding that only 1 in 5 students had good knowledge about FGM/C. Therefore, implementation of the ban in isolation of other interventions is not expected to bring about the rapid and necessary change to achieve FGM/C elimination. The role of television as a media channel in preventing FGM/C was an independent determinant of circumcision of future daughters in the present study. Media coverage, including social media, is important in shifting adolescent girls’ perceptions positively towards discontinuation of FGM/C in Egypt and other African countries [13, 23].

Gender differences in knowledge about FGM/C were not obvious in this sample of university students. This highlights the importance of investing not only in female but also in male education about FGM/C, because men are also affected by its complications; the issue no longer pertains to women alone [24]. Men may play a key role in advocacy as husbands, religious, and community leaders to dismantle the myths about ‘FGM/C makes females happier in their marital lives’ or ‘FGM/C has religious basis’ that were independent determinants of circumcision of future daughters in the present study. Mothers were reported as the main decision-makers for circumcision of their daughters [10]. This may be because half of the women interviewed in the latest Egyptian Demographic Health Survey believed that men prefer the practice to continue [8]; while in fact, recent evidence from Egypt [25] and from some African countries [5] suggests men oppose the practice even more than women. Therefore, men’s collaboration with women in community programs may help initiate a societal dialogue about their actual preferences and roles in this decision.

The level of knowledge about FGM/C in the present study was significantly higher among students with higher parental education. Higher parental education was negatively associated with the prevalence of FGM/C among schoolgirls [10]. Also, the prevalence of FGM/C among daughters of mothers who completed secondary/higher education is half that among daughters of mothers who only completed primary/some secondary education [8]. Positive perceptions towards the discontinuation of FGM/C were significantly higher in females, urban residents, and students with higher parental education, as similarly reported in previous studies [8, 17, 18].

### Strengths and limitations

This study sought to understand university students’ views about FGM/C. Although important, to our knowledge, this subpopulation’s views have been scarcely studied globally and locally [14–18]. A considerable number (2.7 million) of school graduates continued studies in public universities in Egypt in 2017 and this number is likely to increase [26]. University students will presumably have leading and influential roles in the community. The dynamic engagement of youth in this issue is vital, specifically in a country where FGM/C has been banned more than a decade ago, however, a wide gap exists between the aspired and the actual impact of the ban and the other anti-FGM/C interventions on the FGM/C prevalence trends. Also, we have included males and non-medical individuals; relevant views of these subpopulations have been scarcely studied, although they form a majority in the Egyptian society. Furthermore, this may be the first study to assess the awareness of the ban. However, the study sample may not accurately represent the wider views of private and public university students or the general population in Egypt and the cross-sectional design may not allow causal inferences. Random sampling was used to minimize sampling bias. The self-administered method had several advantages considering the sensitivity of the topic: First, it minimized social desirability bias in respondents’ answers that may have resulted from face-to-face interviews. Second, it reduced the possibility of selection bias, where students with specific views about FGM/C may have systematically refused to participate if we have used face-to-face interviews. Third, it diminished interviewer bias; students were given their own space to freely answer the questions without interference or being affected by the gender of the interviewer. No questions about personal experiences of FGM/C were included and we cannot assess how this may have affected female students’ responses; we noted in the pretest the students’ busy schedule and the minimal time they had between classes, therefore, we designed the questionnaire to be simple, short and non-invasive as much as possible to ensure complete and honest responses. Also, the knowledge items in the questionnaire did not cover knowledge about psychological or physical complications to the girl or the mother, such as anxiety, pain, bleeding, mortality, etc. Thus, these items may need to be addressed in future studies. Furthermore, the target sample size has been achieved and we have taken into account non-response and missing values (another 2% was added to the calculated sample based on the pretest results), thus the minimal missing data and possible differences between responders and nonresponders have unlikely biased our findings.

## Conclusions

Understanding youth’s knowledge and perceptions about FGM/C in general and in future daughters in particular was an essential first step that provided insight into the factors that might be hindering the impact of the anti-FGM/C interventions, such as the insufficient knowledge about FGM/C and its ban, and the persistent misperception about FGM/C as a religious percept. Also, this study highlighted opportunities for potential improvement, such as maximizing the use of university curricula and health education sessions – as the main source of knowledge about FGM/C among university students – to increase the anti-FGM/C attitudes among students with neutral perceptions about the practice.

## Supplementary information

**Additional file 1: Supplementary Table 1**. Participant characteristics by faculty.

## Data Availability

All data generated or analysed during this study are included in this published article.

## Abbreviations

FGM/C: Female genital mutilation/cutting
ORa: Adjusted odds ratios
CI: 95% confidence intervals
UK: United Kingdom
USA: United States of America
