# Supplementary material for: Determinants of disagreement with female genital mutilation/cutting of future daughters and awareness of the ban among Egyptian university students
Source: Reprod Health. 2020 Jun 10;17:91. doi: 10.1186/s12978-020-00941-8 (PMC7288485; doi:10.1186/s12978-020-00941-8)
Supplement: Supplementary file 1 — Additional file 1: Supplementary Table 1. Participant characteristics by faculty. [file 12978_2020_941_MOESM1_ESM.docx]

**Supplementary Table 1. Participant characteristics by faculty**

|  | **Total**  N=502 | **Medical** | | | **Non-medical** | | | |
| --- | --- | --- | --- | --- | --- | --- | --- | --- |
|  |  | N=211 | | | N=291* | | | |
|  |  | **Medicine** | **Pharmacy** | **Nursing** | **Law** | **Commerce** | **Engineering** | **Science** |
|  |  | n=49 | n=78 | n=84 | n=51 | n=117 | n=59 | n=63 |
| **Gender*** | 499 | n (%) | n (%) | n (%) | n (%) | n (%) | n (%) | n (%) |
| Male | 270 | 16 (33.3) | 47 (60.3) | 43 (51.2) | 23 (45.1) | 61 (52.1) | 37 (62.7) | 42 (67.7) |
| Female |  | 32 (66.7) | 31 (39.7) | 41 (48.8) | 28 (54.9) | 56 (47.9) | 22 (37.3) | 20 (32.3) |
| **Residence*** | 501 |  | | | | | | |
| Rural | 292 | 27 (55.1) | 52 (66.7) | 46 (54.8) | 29 (56.9) | 67 (57.3) | 37 (62.7) | 34 (54.0) |
| Urban | 209 | 22 (44.9) | 26 (33.3) | 38 (45.2) | 22 (43.1) | 50 (42.7) | 22 (37.3) | 29 (46.0) |
| **Mother's education*** | 500 |  |  |  |  |  |  |  |
| Secondary complete/higher | 236 | 33 (67.3) | 35 (44.9) | 42 (50.0) | 21 (41.2) | 60 (51.3) | 28 (47.5) | 17 (27.4) |
| Primary complete/some secondary | 201 | 13 (26.5) | 34 (43.6) | 34 (40.5) | 22 (43.1) | 46 (39.3) | 21 (35.6) | 31 (50.0) |
| No education/some primary | 64 | 3 (6.2) | 9 (11.5) | 8 (9.5) | 8 (15.7) | 11 (9.4) | 10 (16.9) | 14 (22.6) |
| **Father's education*** | 497 |  | | | | | | |
| Secondary complete/higher | 265 | 37 (75.5) | 38 (48.7) | 44 (53.7) | 22 (43.1) | 66 (57.4) | 33 (55.9) | 24 (38.1) |
| Primary complete/some secondary | 209 | 12 (24.5) | 37 (47.4) | 36 (43.9) | 24 (47.1) | 44 (38.3) | 22 (37.3) | 34 (54.0) |
| No education/some primary | 24 | 0 | 3 (3.9) | 2 (2.4) | 5 (9.8) | 5 (3.5) | 4 (6.8) | 5 (7.9) |

***** Some variables had missing values
